# A data-driven approach to identify risk profiles and protective drugs in COVID-19

**DOI:** 10.1073/pnas.2016877118

**Published:** 2020-12-28

**Authors:** Pietro E. Cippà, Federica Cugnata, Paolo Ferrari, Chiara Brombin, Lorenzo Ruinelli, Giorgia Bianchi, Nicola Beria, Lukas Schulz, Enos Bernasconi, Paolo Merlani, Alessandro Ceschi, Clelia Di Serio

**Affiliations:** ^a^Department of Medicine, Division of Nephrology, Ente Ospedaliero Cantonale, 6500 Bellinzona, Switzerland;; ^b^Faculty of Medicine, University of Zurich, 8006 Zurich, Switzerland;; ^c^University Centre of Statistics in the Biomedical Sciences, “Vita-Salute San Raffaele” University, 20132 Milan, Italy;; ^d^Biomedical Faculty, Università della Svizzera Italiana, 6900 Lugano, Switzerland;; ^e^Clinical School, University of New South Wales, Sydney, NSW 2052, Australia;; ^f^ICT (Informatica e Tecnologia della Comunicazione), Ente Ospedaliero Cantonale, 6500 Bellinzona, Switzerland;; ^g^Department of Medicine, Division of Infectious Diseases, Ente Ospedaliero Cantonale, 6500 Bellinzona, Switzerland;; ^h^Faculty of Medicine, University of Geneva, 1205 Geneva, Switzerland;; ^i^Department of Critical Care Medicine, Ente Ospedaliero Cantonale, 6500 Bellinzona, Switzerland;; ^j^Institute of Pharmacology and Toxicology, Ente Ospedaliero Cantonale, 6500 Bellinzona, Switzerland;; ^k^Department of Clinical Pharmacology and Toxicology, University Hospital Zurich, 8091 Zurich, Switzerland

**Keywords:** COVID-19, survival tree, Bayesian network, RAAS

## Abstract

As the COVID-19 pandemic is spreading around the world, increasing evidence highlights the role of cardiometabolic risk factors in determining the susceptibility to the disease. The fragmented data collected during the initial emergency limited the possibility of investigating the effect of highly correlated covariates and of modeling the interplay between risk factors and medication. The present study is based on comprehensive monitoring of 576 COVID-19 patients. Different statistical approaches were applied to gain a comprehensive insight in terms of both the identification of risk factors and the analysis of dependency structure among clinical and demographic characteristics. The severe acute respiratory syndrome coronavirus 2 (SARS-CoV-2) virus enters host cells by binding to the angiotensin-converting enzyme 2 (ACE2), but whether or not renin−angiotensin−aldosterone system inhibitors (RAASi) would be beneficial to COVID-19 cases remains controversial. The survival tree approach was applied to define a multilayer risk stratification and better profile patient survival with respect to drug regimens, showing a significant protective effect of RAASi with a reduced risk of in-hospital death. Bayesian networks were estimated, to uncover complex interrelationships and confounding effects. The results confirmed the role of RAASi in reducing the risk of death in COVID-19 patients. De novo treatment with RAASi in patients hospitalized with COVID-19 should be prospectively investigated in a randomized controlled trial to ascertain the extent of risk reduction for in-hospital death in COVID-19.

## Significance

The global outbreak of COVID-19 infections generated an unprecedented need to develop novel therapeutic strategies. The SARS-CoV-2 virus enters host cells after binding to the angiotensin-converting enzyme 2 (ACE2), but whether renin−angiotensin−aldosterone system inhibitors (RAASi) are beneficial remains controversial. Standard statistical approaches may fail in assessing medications effects, due to multiple sources of bias in COVID-19 case series collected on an emergency basis. We present a data-driven approach to tackle these challenges. Multilayer risk stratifications were derived for assessing drugs effect, while Bayesian networks were estimated, to analyze dependencies among risk factors’ and treatments’ impact on survival. We provide strong evidence for protectivity of RAASi on hospitalized patients that call for randomized controlled trials of RAASi as COVID-19 treatment option.

The need of discovering rapidly new findings on COVID-19 medications has been rushing publication, whereas the type of data collected in emergency needed a further degree of caution in data management and analysis with respect to the usual observational designed studies. Hence, in the recent literature, standard statistical approaches often failed to provide reliable and reproducible results and to control for the highly correlated structure among covariates, while accounting for potential confounders in risk prediction (1).

COVID-19 is characterized by highly variable clinical manifestations and severity, ranging from asymptomatic to multiorgan failure (2, 3). Older age and cardiovascular comorbidities are among the most important risk factors influencing the virus−host interaction and the clinical outcome of severe acute respiratory syndrome coronavirus 2 (SARS-CoV-2) infection (4–6). Understanding the relationship between cardiovascular disease (CVD), therapy, and COVID-19 outcomes is important to guide clinical and public health interventions. Several treatment approaches, also in light of previous comorbidities, have been adopted to reduce COVID-19 mortality in hospitalized patients. Among other medications, renin−angiotensin−aldosterone system inhibitors (RAASi) have been a major object of interest (7). There are two major arms of RAAS; one arm, the Angiotensin II (Ang II) type 1 receptor (AT1R) pathway, is proinflammatory and can cause acute lung injury (8). The other arm, the angiotensin-converting enzyme 2 (ACE2)−Ang-(1–7)−Mas receptor (MasR) pathway is anti-inflammatory because ACE2 metabolizes Ang II, thus reducing its levels and converting it to the anti-inflammatory peptide, Ang-(1–7) (9). ACE2 is the receptor for coronaviruses, including SARS-CoV-2 (10). When SARS-CoV-2 binds to ACE2, the enzyme is no longer functional, and therefore the proinflammatory Ang II-AT1R is no longer blocked by the ACE2−Ang-(1−7)−MasR pathway; this imbalance can cause acute lung injury (11). RAASi have been hypothesized to influence the clinical course of COVID-19 because of the role of ACE2 as a functional receptor for the virus entrance into the cells (12–14). Initially, some authors raised concerns regarding the potential harm of RAASi in COVID-19, but these were not confirmed, and, more recently, a potential protective role was postulated, but until now not unequivocally proven (11, 15–18).

## Results

At the *Ente Ospedaliero Cantonale* COVID-19−dedicated hospital, we implemented a systematic monitoring, of all admitted COVID-19 patients, that included a predefined set of standardized clinical and laboratory parameters. The study population consisted of 576 hospitalized eligible patients admitted between March 1, 2020 and May 1, 2020. Diagnosis of COVID-19 was based on a positive nasopharyngeal swab specimen tested with real-time RT-PCR assay or high clinical suspicion (as defined in *Materials and Methods*). Demographic and clinical characteristics of patients are shown in Table 1. Crude and adjusted hazard rates were estimated by univariate and multivariate Cox regression analysis in order to identify significant predictors of in-hospital death. In a univariate model age, history of cancer, CVD, chronic kidney disease (CKD) as assessed by the estimated glomerular filtration rate (eGFR), pneumonia on admission, and the prescription of nonsteroidal antiinflammatory drugs (NSAIDs) were associated with increased risk of death. Fever at presentation and the use of RAASi were associated with reduced risk of death (*SI Appendix*, Table S1). Of the 576 patients, 436 had complete records on all variables and were therefore included in a multivariate model. Older age, history of cancer, CVD, and reduced renal function variables were associated with a higher risk of death. Therapy with NSAIDs and with antidiabetic agents was also associated with an increased risk of mortality, whereas RAASi were associated with a markedly reduced risk of death (hazard ratio [HR], 0.34; 95% CI, 0.19 to 0.63; *P* < 0.001), as was therapy with anticoagulants (HR, 0.29; 95% CI, 0.12 to 0.72; *P* = 0.008) (*SI Appendix*, Table S2). To better understand the impact of RAASi, within a multivariate regression framework, the effects of the two main classes of RAASi, angiotensin converting enzyme inhibitor (ACEi) and angiotensin II receptor blocker (ARB), have been also estimated. Both ACEi (HR, 0.45; 95% CI, 0.20 to 0.99; *P* = 0.0474) and ARB (HR, 0.28; 95% CI, 0.13 to 0.61; *P* = 0.0011) were shown to have a significant protective effect.

**Table 1.** Demographic and clinical characteristics of patients

Characteristics (*n* = 576)		Overall
Female sex – no. (%)		218 (37.8)
Age, y		72.0 [60.0, 80.0]
BMI, kg/m^2^ (*n* = 439)		27.6 [24.4, 30.9]
BMI category – no. (%)		
Normal		121 (27.6)
Obese		146 (33.2)
Overweight		165 (37.6)
Underweight		7 (1.6)
Coexisting conditions – no. (%)		
Cancer		65 (11.3)
Diabetes		139 (24.1)
Hypertension		272 (47.2)
CVD		205 (35.6)
Chronic lung disease		96 (16.7)
CKD		197 (34.2)
eGFR category – no. (%)		
<30		43 (7.5)
30 to 60		154 (26.9)
60 to 90		252 (44.0)
≥90		124 (21.6)
Presenting signs and symptoms – no. (%)		
Pneumonia		306 (53.1)
Fever		450 (78.1)
Cough		371 (64.4)
Respiratory symptoms		282 (49.0)
Diarrhea		104 (18.1)
Intensive care – no. (%)		118 (20.5)
Outcome – no. (%)		
Discharged		425 (73.8)
Dead		112 (19.4)
Not discharged		39 (6.8)

Values with [ ] are median and interquartile range, Cases of COVID-19 were diagnosed between March 1 and May 1, 2020.

**Fig. 1.**
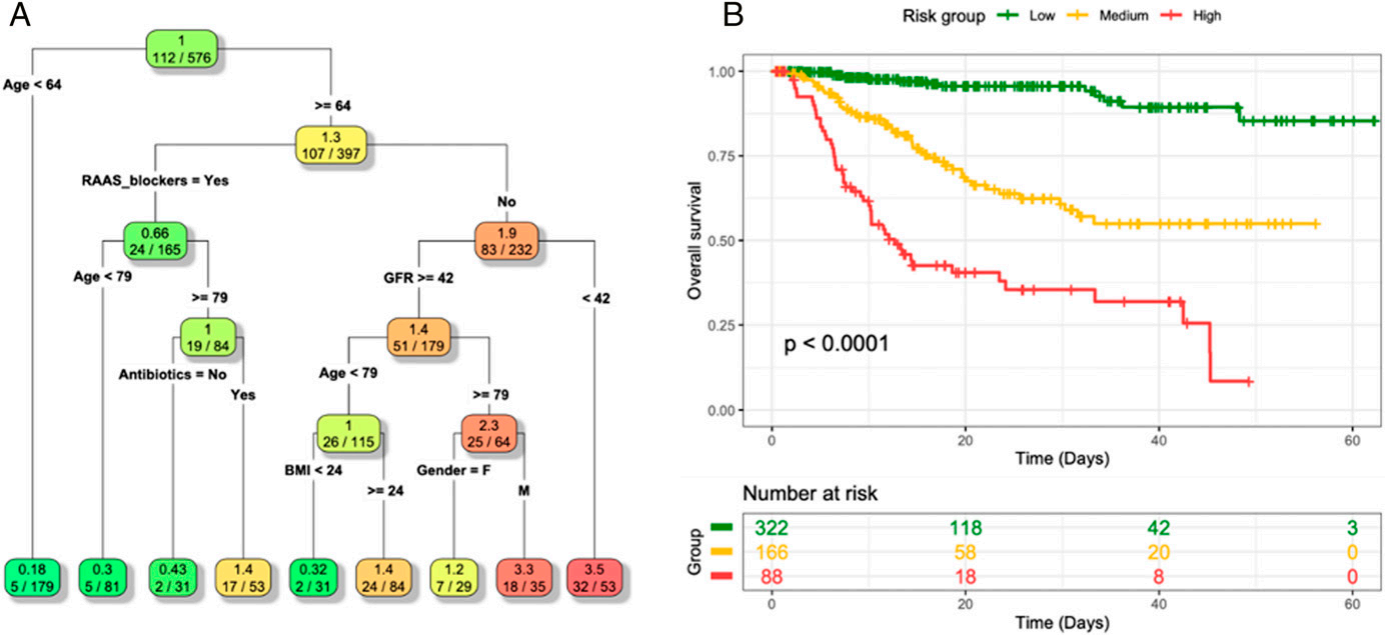
ST analysis (*A*) and Kaplan−Meier curves and log-rank test (*B*) for the three risk groups obtained from the ST analysis based on their HR computed in the final nodes. The low-risk group includes those patients falling in final nodes with an HR lower than 1 (*n* = 322, 63.5%), patients in the medium-risk group are those with an HR between 1 and 2 (*n* = 166, 21.6%), and patients in the high-risk groups are those with an HR higher than 2 (*n* = 88, 16.8%). The *P* value associated with the log-rank test is also displayed.

To uncover natural and homogeneous groups of subjects with similar survival outcome, we applied survival tree (ST) analysis that considered all baseline characteristics and medications at admission and during the hospitalization. The ST analysis selected age, body mass index (BMI), renal function, and treatment with RAASi and antibiotics as split variables, identifying nine risk profiles (Fig. 1*A*). The main discriminant was age, with a favorable outcome in patients ≤64 y old. RAASi were associated with a favorable outcome in patients >64 y old: The individual risk was 0.66 in patients taking and 1.9 in those not taking RAASi. Among patients >64 y old and taking RAASi, patients >79 y old were found to be at higher risk of death, and this risk increased further if an antibiotic therapy was required during the hospitalization. Among patients >64 y old and not taking RAASi, the factor associated with the highest mortality risk was renal function: Those with an eGFR of <42 mL/min per 1.73 m^2^ at baseline displayed a 3.5-fold increased risk of death compared to the rest of the cohort. Among patients aged between 64 and 79 y old and not taking RAASi and with an eGFR of ≥42 mL/min per 1.73 m^2^, BMI of ≥24 kg/m^2^ was strongly associated with death. Thus, the ST analysis identified the different patterns of metabolic and pharmacological risk profiles associated with different clinical outcomes. Based on the HR in the final nodes, leaves were grouped to obtain three risk stratification categories: HR lower that 1 (*n* = 322, 63.5%), HR between 1 and 2 (*n* = 166, 21.6%), and HR higher than 2 (*n* = 88, 16.8%), showing a marked difference in terms of survival (*P* < 0.001; Fig. 1*B*). Furthermore, the ST analysis highlighted the role of critical variables in the context of a multifactorial risk profile: The role of RAASi was particularly intriguing because of their beneficial effect particularly in patients at high risk (age of >64 y).

**Fig. 2.**
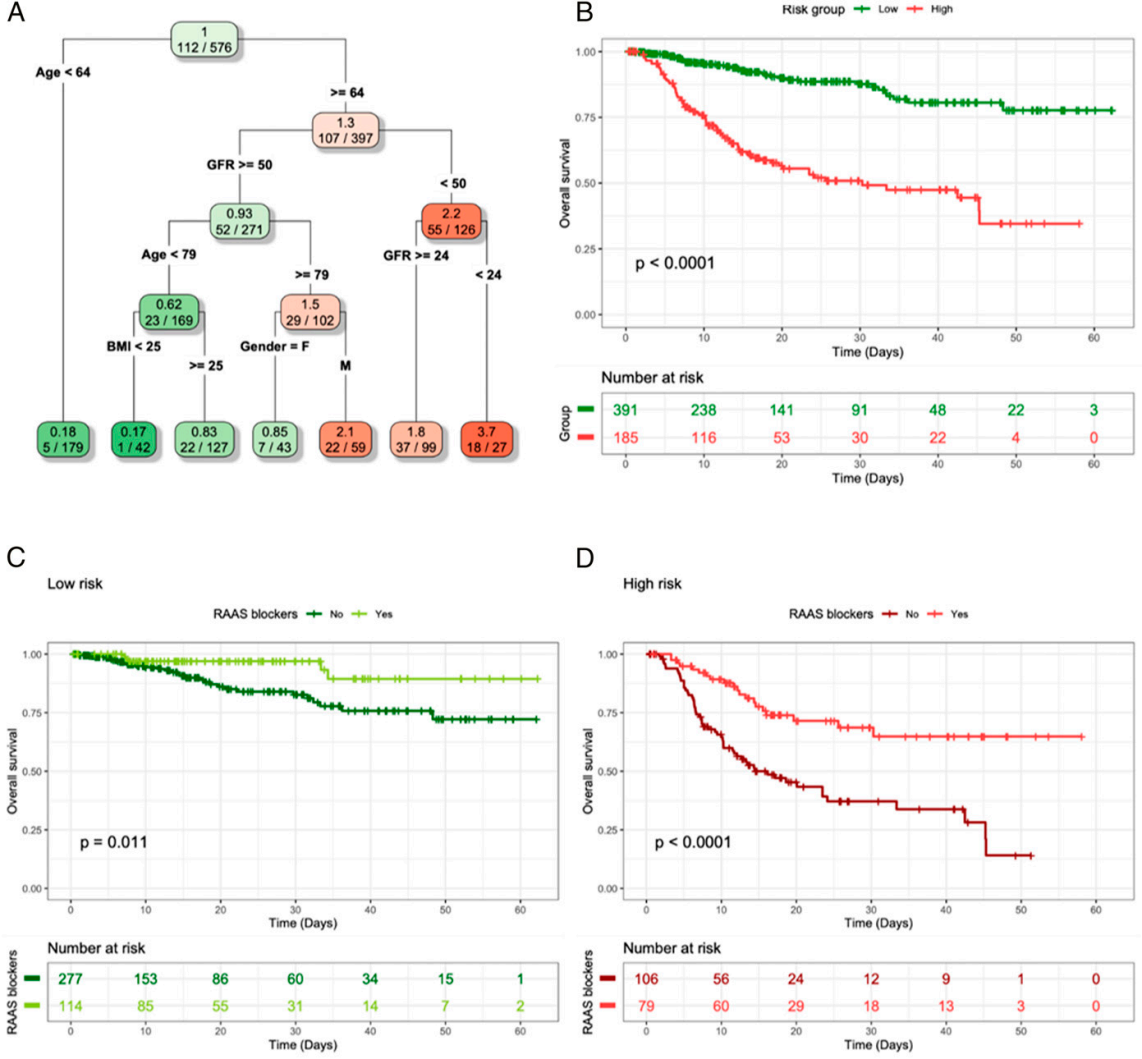
ST analysis without medications as input variables (*A*) and Kaplan−Meier curves and log-rank test (*B*) for the two risk groups obtained from the ST analysis based on their HR computed in the final nodes. The low-risk group includes those patients falling in final nodes with an HR lower that 1 (*n* = 391, 67.8%), and patients in the high-risk groups are those with an HR higher than 2 (*n* = 185, 32.12%). The *P* value associated with the log-rank test is also displayed. Kaplan−Meier curves and log-rank test for the low-risk group (*C*) and the high-risk group (*D*) identified by the ST analysis without medications as input variables. Analysis aimed at examining the differences between patients treated with and without RAAS blockers in the two risk groups. The *P* value associated with the log-rank test is also displayed.

To further validate the role of RAASi, we investigated whether a risk profile built on baseline characteristics only and without treatments still produced risk groups consistent with the protective impact of RAASi. Within the new risk groups with different in-hospital mortality (Fig. 2 *A* and *B*), the association between medication at admission or during hospitalization and in-hospital mortality was evaluated by means of Cox analysis. RAASi were associated with a reduced risk of death (HR 0.34, 95% CI 0.19 to 0.63, *P* < 0.001), with a significant association in both risk stratification groups (Fig. 2 *C* and *D*). In contrast, NSAIDs were associated with a higher in-hospital mortality (HR, 5.18; 95% CI, 2.77 to 9.72; *P* = 0.002; see *SI Appendix*, Fig. S1). We did not find any association between therapy with hydroxychloroquine and mortality.

**Fig. 3.**
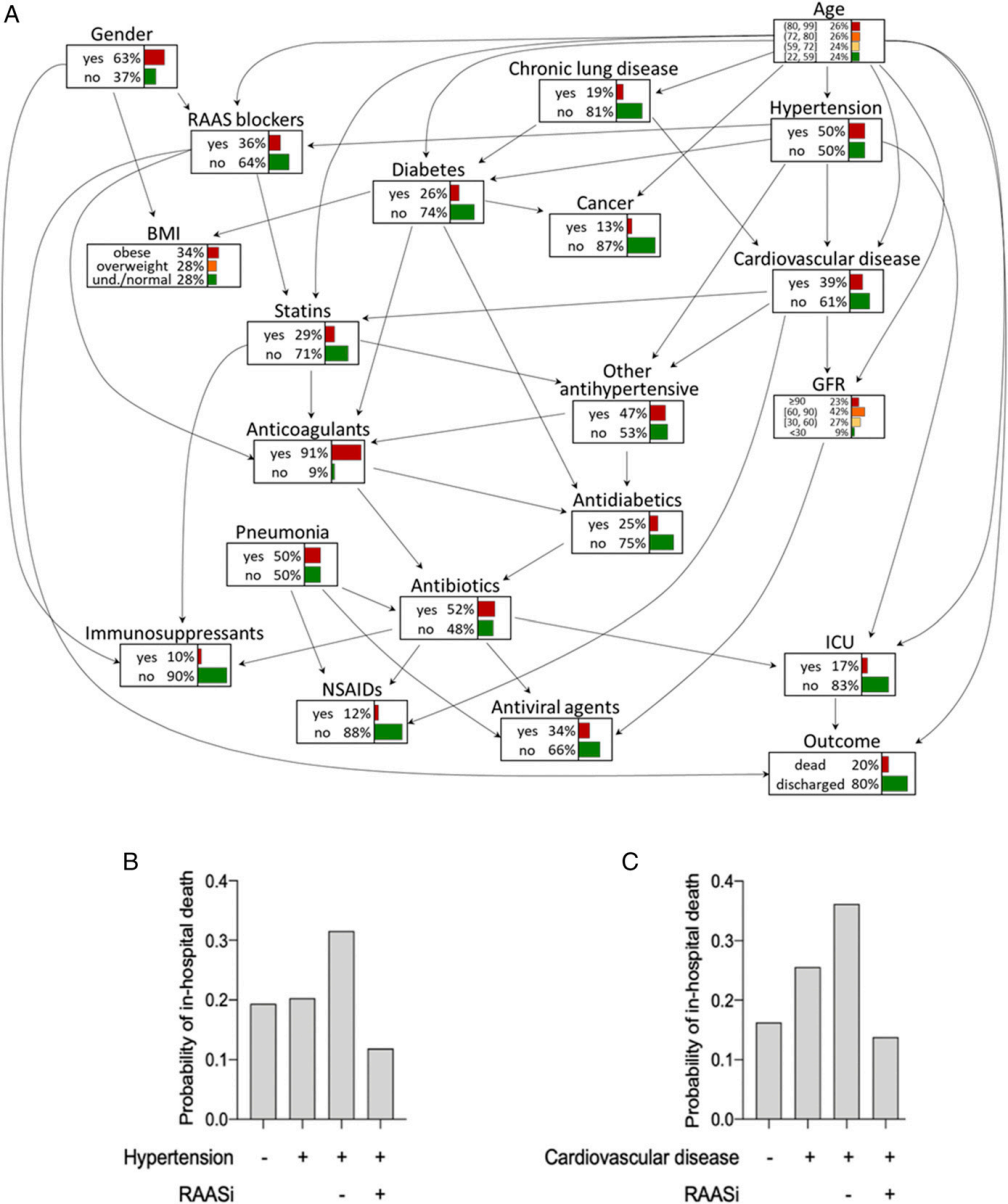
Bayesian network analysis (*A*) exploring the dependence structure of the data. Conditional probabilities of the target variable (in-hospital death) given several scenarios by fixing hypertension and RAASi (*B*) and CVD and RAASi (*C*).

To further investigate the relationships among COVID risk factors and disease progression, Bayesian networks (BNs) were implemented to explore the multivariate dependence structure. The estimated BN is presented in Fig. 3*A*; for each node, the table with estimated marginal probabilities is shown. RAASi, age, and admission to the intensive care unit (ICU) showed a direct effect on the outcome (direct edge). Hypertension showed a direct effect on the admission to ICU and an indirect effect mediated by RAASi and admission to ICU on the outcome (Fig. 3*A*). The key feature of the BN is that it provides the opportunity to evaluate alternative, hypothetical scenarios. By fixing the values of some nodes, it is possible to investigate how a potential change in one crucial variable propagates to all other variables and, in particular, to the target variable. Mortality did not substantially change by modulating the variable hypertension only, but the probability of death decreased in hypertensive patients from 30 to 12% when we fixed the treatment with RAASi (Fig. 3*B*). Similar results were obtained when any CVD was considered instead of hypertension (Fig. 3*C*). The analysis of the relationships between hypertension, CVD, RAASi, and other antihypertensives showed that the estimated probability of death for hypertensive patients treated with RAASi was equal to 10.9%, whereas, for those treated only with other antihypertensive medications, it was 33.2% (*SI Appendix*, Table S3). Similarly, in patients with CVD treated with other antihypertensive agents, the probability of death was 36.8% compared to 13.4% in those treated with RAASi (*SI Appendix*, Table S4).

## Discussion

Our investigation confirms previous reports about the strong association of older age, CVD, and CKD with death in COVID-19 (15, 19).

Additionally, by integrating different methodological approaches, we show that RAASi appear to be highly protective in patients infected with SARS-CoV-2 at increased risk of in-hospital death. In contrast to other studies that used datasets collected on an emergency basis, with a high degree of heterogeneity and highly correlated covariates, we took advantage of a smaller but comprehensive dataset to specifically characterize the interactions between cardiovascular risk factors, medication, and outcome, by means of an integrated statistical approach. This highlighted the need to consider a combination of variables to accurately define the individual risk profile, as exemplified by the cutoff to define the age at risk, which depended on comorbidities, likely reflecting the well-known but hardly measurable difference between chronological and biological age. The multilayer definition of risk defined by the ST analyses (with and without medication) represents a framework to implement the principles of precision medicine in the management of the COVID-19 pandemic, and can be used to identify patients at risk in the context of clinical trials or public health interventions.

In a circumstance characterized by sparse data of low quality, as in most COVID-19 case series, and of disease outcomes strongly affected by confounding bias, obtaining reproducible results to discern decision-making priorities in medication is challenging. We implemented an accurate learning process by approaching different scientific questions with different modeling strategies and by checking for consistency and reproducibility of the conclusion. Different statistical approaches implemented to explore the role of monitored demographic and clinical characteristics on COVID-19 progression not only confirmed the well-known strong association between older age, CVD, and death in COVID-19 (19, 20) but also introduced a rigorous and innovative way to study the impact of medication on survival outcomes in a complex, real-life scenario. The integration and modeling of clinical and pharmacological information provided a different perspective for the evaluation of interconnected variables and introduced flexible models to assess, under different scenarios, the effect of commonly used drugs after SARS-CoV-2 infection. Notably, in line with some recent studies (21), our approach confirmed the lack of beneficial effects of hydroxychloroquine in terms of mortality. The most important findings, from a clinical perspective, are related to the deleterious effect of NSAIDs, previously only inferred from pharmacovigilance observations, and, most importantly, the protective effect of RAASi. Since cardiovascular risk factors, baseline characteristics, and medications are highly associated, with potential confounders dramatically affecting results, a conventional statistical approach may not be appropriate to investigate these complex interdependences. These methodological limitations may explain the controversial findings reported to date on the role of RAASi in COVID-19 (20, 22). Indeed, the beneficial effect of RAASi has been masked by the high dependence on cardiovascular risk factors in previous studies and might reflect a direct impact of RAASi on the virus−host interaction, as previously observed in experimental models of SARS-CoV−induced lung injury displaying a protective role of blocking the RAAS (11, 18). Because ACEi and ARB block two distinct pathways of the RAAS activity, it could be assumed that, when SARS-CoV-2 binds to ACE2, the conversion of Ang II to Ang-(1–7) by ACE2 is impaired, leading to an unhinged proinflammatory effect of Ang II. On one hand, ACEi works by inhibiting the activity of ACE1, therefore blocking the formation of the active peptide Ang II, which is responsible for most actions of the RAAS. On the other hand, ARBs block the interaction of Ang II with the AT1R and cause a rise in Ang II levels, because of a positive feedback on renin release. However, when the AT1R is blocked by ARBs, Ang II can act via the Ang II type 2 receptor (AT2R) to produce effects similar to those generated by the ACE2−Ang (1–7)−MasR pathway (23), provided the ACE2 activity is not blocked. Interestingly, a separate multivariate analysis of the effect of the two main classes of RAASi in our cohort still demonstrated a protective effect of either ACEi alone or ARBs alone and no differential effect of a head-to-head comparison of the two drugs.

Combining evidence provided by advanced multivariate methods like ST and BN represents a robust methodological frame to gain evidence from the data on the complex relationships among variables in different scenarios that cannot be interpreted with standard statistical models (24, 25), thereby opening opportunities to make use of case series to guide public health interventions and to identify drugs with a potential beneficial effect to be evaluated in precisely designed prospective clinical trials. Such trials should not be limited to hypertensive patients, since the blood pressure-lowering effect of RAASi in subjects with a normal baseline blood pressure is negligible (26).

## Materials and Methods

### Data Source and Collection.

The study was supported by the *Ente Ospedaliero Cantonale* and approved by the Ethical Committee of the Canton of Ticino, Switzerland. Written informed consent was waived because of the urgent need to collect data.

The study population consisted of 576 hospitalized patients admitted between March 1, 2020 and May 1, 2020 that were included in a systematic monitoring of admitted patients. Diagnosis of COVID-19 was based on a positive nasopharyngeal swab specimen tested with real-time RT-PCR assay targeting the envelope (env), nucleocapsid (N), and RNA-dependent RNA polymerase (RdRp) genes of SARS-CoV-2. Patients with a high level of suspicion of COVID-19 who had a negative RT-PCR of the nasopharyngeal swab were considered to have COVID-19 if a low-dose CT scan confirmed bilateral and subpleural areas of ground-glass opacification, consolidation affecting the lower lobes, or both (27). In these patients, the diagnosis was later confirmed using RT-PCR assay from subsequent nasopharyngeal swab specimen, lower respiratory tract aspirates, or fecal sample. During the study period, access to the ICU at our hospital has never been limited.

A systematic monitoring of all hospitalized COVID-19 patients, including standardized clinical assessment and comprehensive blood analyses at admission and every 48 h during hospitalization, was established. The recorded data at the time of admission included the following: age, sex, weight, height, medical coexisting conditions, and signs and symptoms of COVID-19 at the time of admission. All clinical and laboratory parameters were extracted from the electronic medical records after checking for matching, outliers, accuracy, precision, and bias by data managers and clinicians. An accurate coding has been done to distinguish drugs taken by the patients at admission and drugs prescribed during hospitalization. Throughout the admission, prescribed drug therapy was determined by the clinical staff in charge of the patient and was not influenced by the study. Particularly, we did not provide any recommendations regarding the introduction or the withdrawal of RAASi, which were mostly further administered during the hospitalization. Single medications were grouped into the following classes, which were subsequently analyzed: RAASi (including renin inhibitors, ACEi, and ARBs), aldosterone antagonists, other antihypertensive drugs, NSAIDs, acetaminophen, oral antidiabetic drugs, insulins, lipid-lowering drugs, antiplatelets, coumarin anticoagulants, direct acting anticoagulants, antibiotics, corticosteroids, immunosuppressants, hydroxychloroquine, and lopinavir/ritonavir.

### Statistical Methods.

Standard and advanced statistical approaches have been implemented to investigate the role of both demographic and clinical characteristics as risk/protective factors for COVID-19 progression, thus deriving different risk profiles, and to explore and disentangle the complex interrelationships among these variables on the clinical outcome.

Univariate and multivariate Cox regression models were estimated, after testing proportionality assumption, to examine how covariates affect hazard rate and to identify factors that significantly affect risk of death. As previously reported (28), this was considered the most appropriate approach, since COVID-19 is characterized by a time interval (from admission or from first symptom), which would not be considered in a multivariate logistic regression. Demographic characteristics, comorbidities, pneumonia, and drug therapy were entered in the model as covariates, and the results were reported as HR with 95% CIs. Univariate *P* values were adjusted to control the family-wise error rate (29).

To profile subjects with similar survival outcome, ST analysis, a statistical learning approach that builds classification trees with respect to survival endpoints, was applied using, as independent variables, the same covariates entered in the Cox models (30). STs were applied to profile patients with respect to risk of death and to disentangle the role of highly dependent covariates on risk stratification. In a data-driven approach, which generalized standard classification trees (24), the best predictors with the best thresholds are selected by the iterative algorithm to identify homogeneous subgroups of patients. Herein, trees are used in a twofold perspective: exploratory and predictive (31, 32). From one side, we focus on the search for those variables (and corresponding cutoffs) that best discriminate among patients based on their survival outcome. However, trees-based procedures are prone to overfitting. For this reason, in the tree-building phase, we have imposed a constraint by fixing the minimum number of observations in any terminal node at 20. This choice was motivated by the need to have enough observations in the nodes to properly carry out further analyses. Actually, following ST analysis, the Kaplan−Meier method was used to estimate overall survival for each risk profile, and log-rank test has been applied to compare survival among groups of patients defined based on the medications.

Finally, according to a reproducibility and replicability perspective intended as the ability of independent researchers to obtain the same result using different methodological frames, we check for consistency of results among different statistical models: The BN approach was used to evaluate the dependence structure among all variables included in the Cox multivariate model. This is an essential step to uncover complex interrelationships among variables and to gain a better insight into mechanisms involved in COVID-19 disease progression. BNs implement a graphical model structure, known as a directed acyclic graph, defined by a set of nodes, representing random variables, and a set of arcs, implying direct dependencies among the variables. They enable an effective representation and computation of a joint probability distribution over a set of random variables (33).

The purpose of using BN in this research is to learn dependence structure directly from data, while excluding some directions among variables that are not consistent with the very nature of the data. These are the so-called “blacklists” directions, arcs, which are not allowed in the network. In this analysis, we specified only blacklists as illustrated in *SI Appendix*, Fig. S2. Variables included in the same box are free to learn the type of interactions directly from the data. The only relationships among variables in different boxes that are allowed are those indicated by arrows in *SI Appendix*, Fig. S2. Otherwise, relationships among variables in different boxes are forbidden (for example, comorbidities cannot influence demographics characteristics).

The network has been estimated from data by a hill climbing algorithm with Akaike information criterion score functions. On the basis of the estimated network, various diagnostic checks have been performed to investigate the effects of evidence on the distribution of the target variable using “what-if” sensitivity scenarios (34).

All of the analyses were performed using R statistical software (version 3.5.2; https://cran.r-project.org/index.html). The R package rpart was used to implement the ST analysis: The procedure applies the LeBlanc and Crowley splitting rule (35). The R packages bnlearn (36) and gRain (37) were used to learn the network and perform the inference required to calculate the conditional probabilities.

## Supplementary Material

Supplementary File

## Data Availability

Anonymized data have been deposited in The Open Science Framework at https://osf.io/sj4zu. The password to open the file will be provided by the authors upon request.
