# Supplementary material for: A data-driven approach to identify risk profiles and protective drugs in COVID-19
Source: Proc Natl Acad Sci U S A. 2020 Dec 28;118(1):e2016877118. doi: 10.1073/pnas.2016877118 (PMC7817222; doi:10.1073/pnas.2016877118)
Supplement: Supplementary File [file pnas.2016877118.sapp.pdf]

Supplementary Information for

A data-driven approach to identify risk profiles and protective drugs in COVID-19.

Pietro E Cippà M.D. Ph.D.,[a,b,1,2] Federica Cugnata Ph.D.,[c,1] Paolo Ferrari M.D.,[a,d,e,1] Chiara Brombin Ph.D.,[c] Lorenzo Ruinelli M.Sc.,[f] Giorgia Bianchi M.D.,[a] Nicola Beria M.D.,[a] Lukas Schulz M.D.,[a] Enos Bernasconi M.D.,[d,g,h] Paolo Merlani M.D.,[h,i] Alessandro Ceschi M.D. M.Sc.,[b,d,j,k,,1] Clelia Di Serio Ph.D.[c,d,1,2]

[a] Department of Medicine, Division of Nephrology, Ente Ospedaliero Cantonale, 6500 Bellinzona, Switzerland.
[b] Faculty of Medicine, University of Zurich, 8006 Zurich, Switzerland.
[c] University centre of Statistics in Biomedical Sciences, "Vita-Salute San Raffaele" University, 20132 Milan, Italy.
[d] Biomedical Faculty, Università della Svizzera Italiana, 6900 Lugano, Switzerland.
[e] Clinical School, University of New Wales, Sydney, NSW 2052, Australia.
[f] ICT, Ente Ospedaliero Cantonale, 6500 Bellinzona, Switzerland.
[g] Department of Medicine, Division of Infectious diseases, Ente Ospedaliero Cantonale, 6500 Bellinzona, Switzerland.
[h] Faculty of Medicine, University of Geneva, 1205 Geneva, Switzerland.
[i] Department of critical care medicine, Ente Ospedaliero Cantonale, 6500 Bellinzona, Switzerland.
[j] Institute of Pharmacology and Toxicology, Ente Ospedaliero Cantonale, 6500 Bellinzona, Switzerland.
[k] Department of Clinical Pharmacology and Toxicology, University Hospital Zurich, 8091 Zurich, Switzerland.

[1] P.E.C., F.C., P.F. , A.C., C.D.S. contributed equally to this work

[2] To whom correspondence may be addressed. Email: pietro.cippa@eoc.ch and diserio.clelia@hsr.it

This PDF file includes:

Figures S1 to S2
Tables S1 to S4

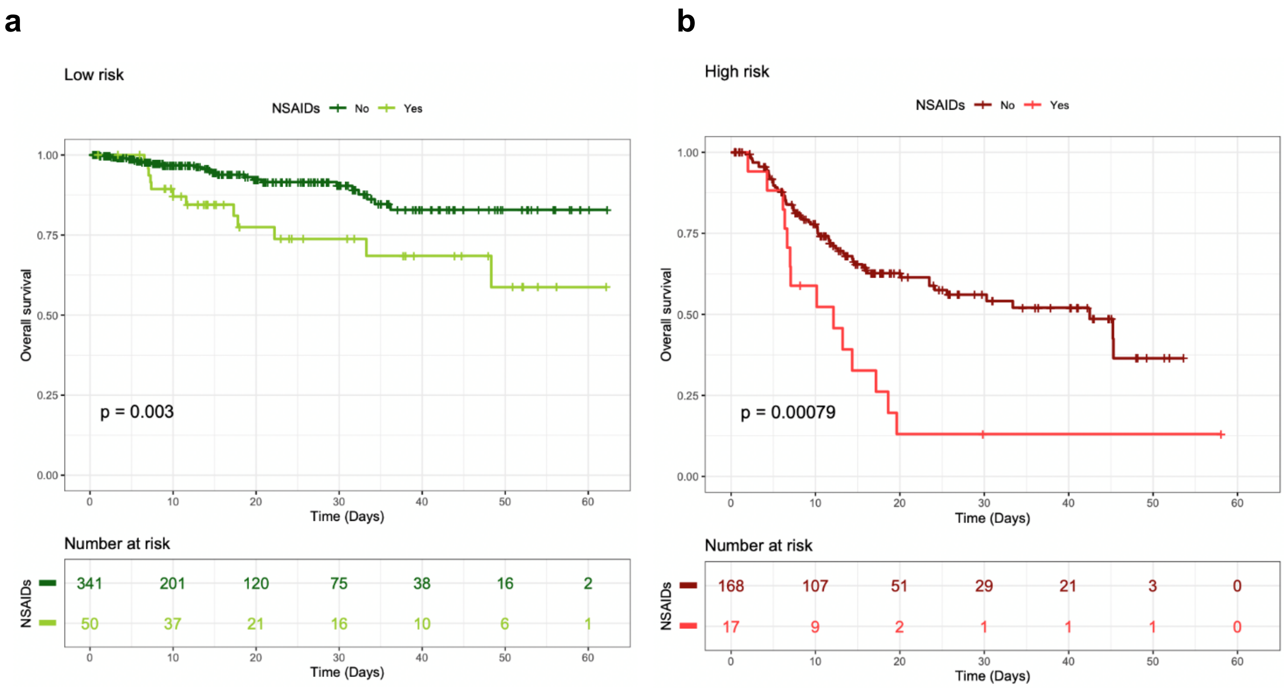

Fig. S1 Survival analysis comparing patients with and without NSAID therapy. Patients at low risk according to the survival tree presented in Figure 2a are shown in panel a, patients at high risk in panel b.

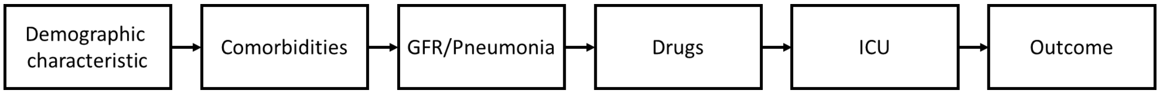

Fig. S2 Logical constraints used in the Bayesian Network estimation. Relationships among variables in different boxes are forbidden (for example comorbidities cannot influence demographics characteristics). Indeed, variables included in the same box are allowed to learn freely the type of interaction with the other variables.

Table S1. Cox's univariate models.

	HR (95% CI)	p-value	adj. p-value
Male sex	1.09 (0.74,1.62)	0.657	1.000
Age	1.06 (1.04,1.07)	<0.001	0.000
BMI	1.02 (1,1.04)	0.036	0.400
ICU	1.09 (0.73,1.64)	0.662	1.000
Cancer	2.4 (1.55,3.73)	<0.001	0.001
Diabetes	1.39 (0.93,2.06)	0.107	0.807
Hypertension	1.25 (0.86,1.81)	0.242	0.989
Cardiovascular disease	2.53 (1.73,3.68)	<0.001	0.000
Chronic lung disease	1.15 (0.72,1.83)	0.564	1.000
Pneumonia	1.46 (0.99,2.15)	0.055	0.567
eGFR	0.98 (0.97,0.98)	<0.001	0.000
RAAS blockers	0.44 (0.28,0.69)	<0.001	0.003
Other antihypertensive agents	0.92 (0.63,1.33)	0.657	1.000
NSAIDs	2 (1.29,3.1)	0.002	0.022
Antidiabetics	1.1 (0.73,1.65)	0.658	1.000
Statins	0.66 (0.43,1.03)	0.067	0.630
Anticoagulants	0.54 (0.33,0.87)	0.012	0.135
Antibiotics	1.43 (0.96,2.13)	0.075	0.668
Immunosuppressants	1.01 (0.56,1.79)	0.986	1.000
Antiviral agents	0.74 (0.49,1.11)	0.143	0.895

Table S2. Cox's multivariate models including all parameters (n=436)

	adj. HR(95%CI)	p-value
Male sex	1.35 (0.8,2.26)	0.262
Age	1.04 (1.02,1.07)	0.002
BMI	1.01 (0.99,1.03)	0.41
ICU	1.00 (0.58,1.72)	0.993
Cancer	1.95 (1.12,3.4)	0.019
Diabetes	0.55 (0.28,1.09)	0.086
Hypertension	0.91 (0.53,1.57)	0.744
Cardiovascular disease	3 (1.62,5.57)	<0.001
Chronic lung disease	1.18 (0.67,2.08)	0.566
Pneumonia	1.24 (0.75,2.04)	0.397
eGFR	0.97 (0.96,0.99)	<0.001
RAAS blockers	0.34 (0.19,0.63)	<0.001
Other antihypertensive agents	0.53 (0.29,0.97)	0.041
NSAIDs	5.34 (2.84,10.04)	<0.001
Antidiabetics	2.31 (1.13,4.71)	0.022
Statins	0.63 (0.35,1.13)	0.123
Anticoagulants	0.29 (0.12,0.72)	0.008
Antibiotics	1.68 (0.93,3.04)	0.084
Immunosuppressants	0.95 (0.48,1.87)	0.882
Antiviral agents	1.65 (0.93,2.92)	0.087

Table S3. Bayesian network analysis. Conditional probabilities of the target variable (in-hospital death) given several scenarios focused on hypertension and drugs.

Scenarios	discharged	dead
Hypertension = no	0.8063	0.1937
Hypertension = yes	0.7970	0.2030
Hypertension = yes; RAAS blockers = no; Other antihypertensive agents = no	0.7128	0.2872
Hypertension = yes; RAAS blockers = yes; Other antihypertensive agents = no	0.8915	0.1085
Hypertension = yes; RAAS blockers = no; Other antihypertensive agents = yes	0.6680	0.3320
Hypertension = yes; RAAS blockers = yes; Other antihypertensive agents = yes	0.8766	0.1234

Table S4. Bayesian network analysis. Conditional probabilities of the target variable (in-hospital death) given several scenarios focused on cardiovascular disease and drugs.

Scenarios	discharged	dead
Cardiovascular disease = no	0.837	0.163
Cardiovascular disease = yes	0.744	0.256
Cardiovascular disease = yes; RAAS blockers = no; Other antihypertensive agents = no	0.649	0.351
Cardiovascular disease = yes; RAAS blockers = yes; Other antihypertensive agents = no	0.866	0.134
Cardiovascular disease = yes; RAAS blockers = no; Other antihypertensive agents = yes	0.632	0.368
Cardiovascular disease = yes; RAAS blockers = yes; Other antihypertensive agents = yes	0.861	0.139
